# Local characteristics of molecular epidemiolgy of *Acinetobacter baumannii* in Jilin province (northeast China)

**DOI:** 10.1186/s12866-023-02761-9

**Published:** 2023-01-19

**Authors:** Qingsong You, Xue Du, Nannan Hu, Yusi Zhang, Na Zhang, Fusheng Wang, Jinghua Li, Yanbo sun, Fang Wang, Hongyan Shi

**Affiliations:** 1 Department of Pathogenobiology, CollegeofBasicMedicalScience, Jilin University, 126, Xinmin Street, Changchun, 130021 Jilin China; 2 First Hospital of Jilin University, 1, Xinmin Street, Changchun, China; 3 Affiliated Hospital of Changchun University of Chinese Medicine, 1478, Gongnong Avenues, Changchun, China

**Keywords:** *Acinetobacter baumannii*, Multi-locus sequence typing, Phylogeny, Geographic region, Epidemiology, Antibiotic resistance

## Abstract

*A. Baumannii* is an opportunistic nosocomial pathogen which has severe antibiotic resistance. However, the epidemiology is less clearly understood in Jilin province and China. Thus, 89 *A. baumannii* isolates from a single hospital in Jilin province between 2013–2017 were performed by MLST. In order to better understanding of the epidemiology of Jilin isolates, Chinese strains originated from other domestic regions and worldwide isolates in MLST database were analyzed by *silico* phylogenetic tools together. A total of 22 STs in Jilin were identified, and 10 STs were found to be novel. The top three predominant sequence types are ST195 (*n* = 34, 38.2%), ST208 (*n* = 14, 15.7%) and ST540 (*n* = 13, 14.6%). ST369 is predicted to be group founder and ST195, ST540 are subgroup founders of the majority STs in Jilin Province. Some newly discovered singletons showed close relationship with strains from other countries, which suggest that nation-cross transmission is one of important origin of Jilin strains. The majority of Jilin STs showed clonality and close relationship with the majorities from other regions of China. But occupation of individual STs in Jilin were different from that of other domestic regions. The aggregation trend and genetic relationship proved that predominant Jilin STs continue to mutate during transmission. Drug resistance facilitated transmission of Jilin *A.baumannii* isolates because more than 94% of isolates are resistant to at least one carbapenem and the STs with strong resistance to carbapenems usually has more isolates. In conclusion, high diversity and different occupation of STs, and occupation of novel STs proved that epidemiology of *A. baumannii* in Jilin has special regional characteristics, and drug resistance facilitated transmission of domestic strains and foreign strains.

## Highlights

1. The epidemiology of *A. baumannii* in Jilin Province showed distinctive local characteristic.

2. Newly discovered sequence types (STs), the results of genetic relatedness and geographical distribution of all STs showed epidemiological research by multi-locus sequence typing (MLST) in single hospital is helpful to monitor and control nosocomial infection of *A. baumannii*.

3. The rate of carbapenems resistance among *A. baumannii* is about 94% in Jilin province (northeast China). Carbapenems resistance is a helpful factor for predominant STs spreading.

## Introduction

The gram-negative bacterium *A. baumannii* is an opportunistic nosocomial pathogen inducing a vast array of infections, of which the most common infections are ventilator-associated pneumonia and bloodstream infections with high mortality rate [1]. Multidrug resistance (MDR) limits therapeutic options and impacts on clinical care. Although carbapenems were considered the mainstay against MDR *A. baumannii* infections, carbapenem resistant *A. baumannii* has been prioritized as the top one of WHO priority pathogens list that are urgent to research and develop new antibiotics [2, 3]. However, infection prevention and control are most effective when applied at the earliest possible stage. Molecular epidemiology is an indispensable tool to early monitor the connection of pathogen clusters with infections, which is also helpful to identify the origin of causative organisms and investigate healthcare-associated infections [4].

MLST is a proven tool for the molecular typing of *A. baumannii*, MLST helps not only in tracking the sequence types (STs) and outbreaks, but also in understanding the microbial evolution [5]. The relevant cluster analysis based on STs also presents several advantages, such as the ease of interpretation and the creation of hierarchical grouping of the isolates. Moreover, it provides a global overview of the relatedness of the isolates and how the defined clusters are connected to each other [6].

In China, carbapenems resistant *A. baumannii* is also on the rise, which has become a serious threat to public health [7]. Some papers on MLST showed that clonal complex 92 (CC92) is the predominant group with the widest distribution in Chinese mainland [8–10]. However, to our knowledge, the strains in these studies were mainly isolated from other regions of China. The epidemiology of *A. baumannii* is less clearly understood in Jilin province as only six Jilin strains with same ST were registered in MLST database before our study. Therefore, STs of 89 *A. baumannii* isolates from a single hospital in Jilin between 2013–2017 were analyzed by MLST and other *silico* phylogenetic tools. For the sake of analyzing regional characteristics, previously registered Chinese isolates and large amount of global collection of *A. baumannii* from MLST database (https://pubmlst.org/organisms/acinetobacter-baumannii) were also covered in this study.

## Results

Among obtained STs, the number of alleles in each locus varied from 5 to 14 and that of SNPs ranged from 5 to 63. The sequence diversity of each locus ranged from 2.98 to 6.40% with average value of 3.83%. Furthermore, nucleotide diversity (π) in this study was in the range of 0.00413 to 0.03514 (Table 1).

**Table 1:** Property of the MLST loci in *A baumannii* in this study

Locus	No. of nucleotide analyzed	No. of alleles	No. of SNP	SNP Frequency	No. of variable sites	Nucleotide diversity π	Sequence diversity rate
Oxf_gltA	484	5	5	1.03%	5	0.00413	2.98%
Oxf_gyrB	457	10	20	4.38%	19	0.01255	3.92%
Oxf_gdhB	344	10	20	5.81%	20	0.01628	3.73%
Oxf_recA	371	11	63	16.98%	60	0.03514	6.40%
Oxf_cpn60	421	7	11	2.61%	11	0.00882	3.87%
Oxf_gpi	305	14	48	15.74%	44	0.03967	3.38%
Oxf_rpoD	513	7	6	1.17%	6	0.00464	3.29%

### Antimicrobial susceptibility

The result of antimicrobial susceptibility is shown in Table 2. Totally, the resistant portions of Jilin isolates to ertapenem, imipenem, meropenem, tigecycline, and colistin B are 91.01% (*n* = 81), 87.64% (*n* = 78), 89.89% (*n* = 80), 7.86% (*n* = 7), and 13.48% (*n* = 12), respectively. Twelve isolates were found to be sensitive to at least one of tested carbapenems. The overwhelming majority in our Jilin isolates (77/89) were resistant to all tested carbapenems, and 75 of them were at least sensitive to one or both of tigecycline and colistin B. But one isolate was resistant to all tested antibiotics including tigecycline and colistin B.

**Table 2:** Isolates study based on the geographical location, ST(MLST(Oxford)), year of isolation, antimicrobial susceptibility

Isolate ID	Geographical location	ST (MLST (Oxford))	Year of isolation	Ertapenem	Imipenem	Meropenem	Tigecycline	Colistin B
6350	Jilin	2370^a^	2017	R	R	R	S	R
6351	Jilin	2393^a^	2017	R	R	R	R	R
6352	Jilin	2395^a^	2017	I	S	S	S	R
6353	Jilin	2396^a^	2017	R	R	S	S	S
6354	Jilin	2397^a^	2017	S	S	S	R	S
6355	Jilin	2398^a^	2017	R	S	R	S	S
6356	Jilin	2399^a^	2017	S	S	S	S	S
6357	Jilin	2400^a^	2017	R	R	R	S	S
6358	Jilin	2401^a^	2017	S	S	S	R	S
6359	Jilin	2402^a^	2017	S	S	S	S	S
6430	Jilin	195	2014	R	R	R	S	S
6431	Jilin	195	2014	R	R	R	S	S
6432	Jilin	195	2014	R	R	R	S	R
6433	Jilin	195	2014	R	R	R	S	R
6434	Jilin	195	2014	S	S	R	S	R
6435	Jilin	195	2014	R	R	R	S	R
6436	Jilin	195	2014	R	R	R	S	S
6437	Jilin	195	2014	R	R	R	S	S
6438	Jilin	195	2014	R	R	R	S	S
6439	Jilin	195	2014	R	R	R	S	S
6440	Jilin	195	2014	R	R	R	S	S
6441	Jilin	195	2014	R	R	R	S	S
6442	Jilin	195	2014	R	S	R	S	S
6443	Jilin	195	2014	R	R	R	S	S
6444	Jilin	195	2014	R	R	R	S	S
6445	Jilin	195	2014	R	R	R	S	S
6446	Jilin	195	2014	R	R	R	S	S
6447	Jilin	195	2017	R	R	R	S	S
6448	Jilin	195	2017	R	R	R	S	S
6449	Jilin	195	2017	R	R	R	S	S
6450	Jilin	195	2017	R	R	R	S	S
6451	Jilin	195	2017	R	R	R	S	S
6452	Jilin	195	2017	R	R	R	S	S
6453	Jilin	195	2017	R	R	R	S	S
6454	Jilin	195	2017	R	R	R	S	S
6455	Jilin	195	2017	R	R	R	S	S
6456	Jilin	195	2017	R	S	S	S	R
6457	Jilin	195	2015	R	R	R	S	S
6458	Jilin	195	2015	R	R	R	S	S
6459	Jilin	195	2015	R	R	R	S	S
6460	Jilin	195	2015	R	R	R	S	S
6461	Jilin	195	2015	R	R	R	S	S
6462	Jilin	195	2015	R	R	R	R	S
6463	Jilin	195	2015	R	R	R	S	S
6464	Jilin	208	2015	S	R	R	S	S
6465	Jilin	208	2015	R	R	R	S	S
6466	Jilin	208	2015	R	R	R	S	S
6467	Jilin	208	2015	R	R	R	S	S
6468	Jilin	208	2015	R	R	R	S	S
6469	Jilin	208	2015	R	R	R	S	R
6470	Jilin	208	2016	R	R	R	S	S
6471	Jilin	208	2016	R	R	R	S	S
6472	Jilin	208	2016	R	R	R	S	S
6473	Jilin	208	2016	R	R	R	S	S
6474	Jilin	208	2016	R	R	R	S	S
6475	Jilin	208	2016	R	R	R	S	S
6476	Jilin	208	2016	R	R	R	S	S
6477	Jilin	208	2016	R	R	R	S	S
6478	Jilin	218	2016	R	R	R	S	S
6479	Jilin	218	2016	R	R	R	S	S
6480	Jilin	540	2016	R	R	R	S	S
6481	Jilin	368	2016	R	R	R	S	R
6482	Jilin	368	2016	R	R	R	R	S
6483	Jilin	368	2013	R	R	R	R	S
6484	Jilin	369	2013	R	R	R	S	S
6485	Jilin	369	2013	R	I	R	S	S
6486	Jilin	373	2013	S	S	S	R	S
6487	Jilin	469	2013	R	R	R	S	S
6488	Jilin	540	2013	R	R	R	S	R
6489	Jilin	540	2013	R	R	R	S	S
6490	Jilin	540	2013	R	R	R	S	S
6491	Jilin	540	2013	R	R	R	S	S
6492	Jilin	540	2014	R	R	R	S	S
6493	Jilin	540	2014	R	R	R	S	S
6494	Jilin	540	2014	R	R	R	S	R
6495	Jilin	540	2014	R	R	R	S	S
6496	Jilin	540	2014	R	R	R	S	S
6497	Jilin	540	2014	R	R	R	S	S
6498	Jilin	540	2014	R	R	R	S	S
6499	Jilin	540	2016	R	R	R	S	S
6500	Jilin	699	2016	S	R	S	S	S
6502	Jilin	1199	2016	R	R	R	S	S
6503	Jilin	1779	2017	R	R	R	S	S
6504	Jilin	1779	2017	R	R	R	S	S
6505	Jilin	1779	2017	R	R	R	S	S
6506	Jilin	1779	2015	R	R	R	S	S
6507	Jilin	1779	2015	R	R	R	S	S
6508	Jilin	1779	2015	R	R	R	S	S
6509	Jilin	1926	2015	R	R	R	S	S

*R* Resistant, *S* Sensitive, *I* Intermediate

^a^Novel STs were found in this study

### MLST analysis

Among 89 Jilin isolates, 22 STs were identified, among them ten STs including ST2370, ST2393, ST2395, ST2396, ST2397 ST2398, ST2399, ST2400, ST2401, and ST2402 were found for the first time. It should be noted, ST1779, with 6 isolates, was not recognized as novel ST in MLST Database, but the clues to the existence of ST1779 was hardly to be founded. The frequencies of STs ranged from 1–34 with ST195 (*n* = 34), ST208 (*n* = 14), ST540 (*n* = 13), ST1779 (*n* = 6), ST368 (*n* = 3), ST218 (*n* = 2) and ST369 (*n* = 2) as the majority. All newly discovered STs just occupied one isolate in this study. The previously registered ST75 was not found in this study.

### The association between Jilin STs and carbapenem resistance

**Fig. 1:**
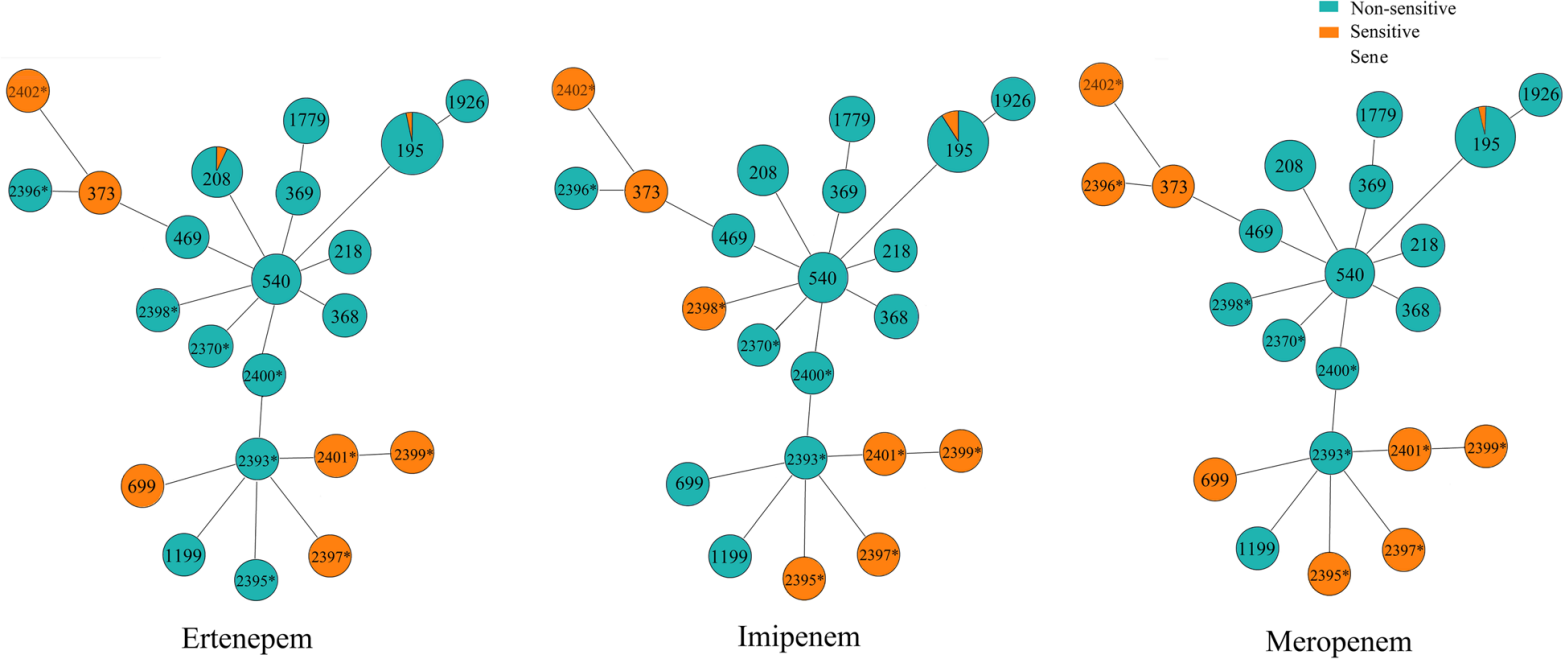
PHYLOViZ Online analysis showing the genetic relationship and three carbapenems antibiotics among Jilin province in this study. Each dot represents a distinct ST. STs in green color are resistant isolates, and STs in orange color are sensitive isolates

ST 195 (*n* = 3), ST373 (*n* = 1), ST699 (*n* = 1), ST2395 (*n* = 1), ST2396 (*n* = 1), ST2397 (*n* = 1), ST2398 (*n* = 1), ST2399 (*n* = 1), ST2401 (*n* = 1), and ST2402 (*n* = 1) were found to be sensitive to at least one of tested carbapenems, and among them just ST195, ST373 and ST699 were previously discovered STs. The occupation of carbapenems sensitive isolates in all previously discovered STs was about 7.6% (6/79), while that in newly discovered STs was 70% (7/10). The attempts have been done to ensure the association between the STs and the antimicrobial susceptibility. In this study, ST373 (*n* = 1), ST699 (*n* = 1), ST2395 (*n* = 1), ST2396 (*n* = 1), ST2397 (*n* = 1), ST2399 (*n* = 1), ST2401 (*n* = 1), ST2402 (*n* = 1) were found to be sensitive to ertapenem, imipenem and meropenem in general, the overwhelming majority isolates of other STs are non-sensitive to three carbapenems antimicrobial, and association was statistically significant (*P* < 0.001, by Fisher’s precision probability test). The association between the STs and the carbapenem sensitivity was showed in Fig. 1.

### Genetic relatedness

ST195, ST208, ST218, ST368, ST369, and ST540, which are predominant STs in Jilin could be classified into CC92 group. However, other main STs in Jilin including ST1779, ST1926, and ST2370, could not be classified into CC92 group because of lacking sufficient alleles of the loci in housekeeping genes. It must be pointed out that although ST1779 (*n* = 6) is not a novel ST, it has never been founded in MLST database before.

**Fig. 2:**
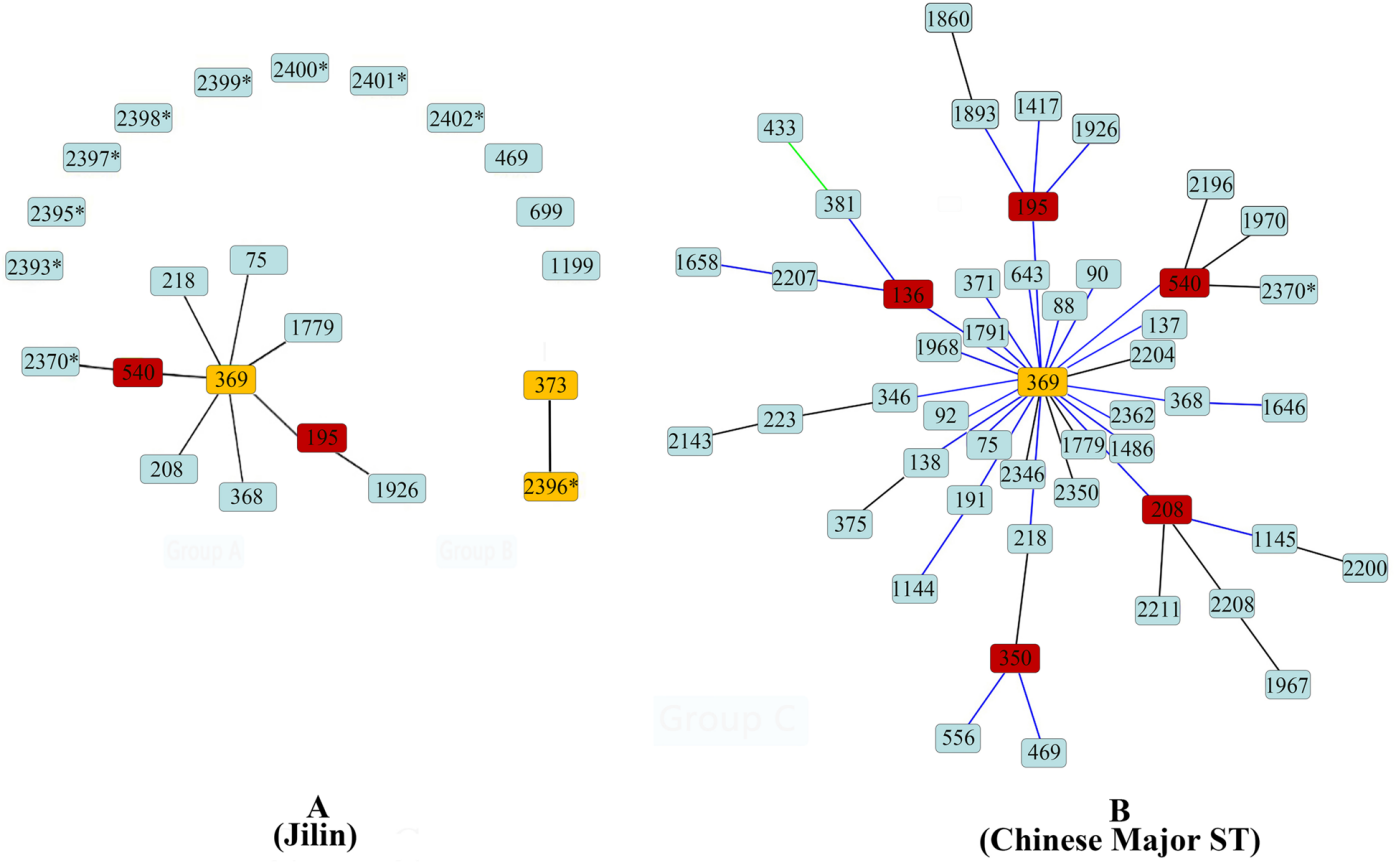
Group **A** (Jilin): genetic relationship of *A. baumannii* isolates of Jilin province using goeBURST (ST75 is obtained from MLST database, other 22 STs are obtained in this study, * refers to novel ST that is found in this study). And Group **B** (Chinese Major ST): genetic relationship of the majority *A. baumannii* isolates of China using goeBURST. Yellow node refers to group founder, red node refers to subgroup founder, light blue refers to common node

The goeBURST analysis within the scope of Jilin isolates showed that 78 our isolates in nine STs and six previous isolates in ST75 could be classified into a big group with ST369 as group founder and ST195, ST540 as subgroup founders (Fig. 2A). And among 10 novel STs, 8 newly identified STs are singletons without any association with other Jilin isolates (Fig. 2A). The details of locus variation in goeBURST algorithm were displayed in Table 3.

**Table 3:** Description of STs based on goeBURST analysis and the earliest year of occurrence in Jilin province

ST	Frequency	SLV	DLV	TLV	Satellite	Earliest year of occurrence	
195a	34	6	2			2014	
208^a^	14	5	3			2015	
218^a^	2	5	3			2016	
368^a^	3	5	3			2013	
369^a^	2	6	2			2013	
540^a^	13	6	2			2013	
1779^a^	6	1	5	2	2	2015	
1926^a^	1	1	5	2	2	2015	
2370^a^	1	1	5	2	2	2017	
373^b^	1	1				2013	
2396^b^	1	1				2017	
469^c^	1					2013	
699^c^	1					2016	
1199^c^	1					2016	
2393^c^	1					2017	
2395^c^	1					2017	
2397^c^	1					2017	
2398^c^	1					2017	
2399^c^	1					2017	
2400^c^	1					2017	
2401^c^	1					2017	
2402^c^	1					2017	

^a^STs of Group A in ‘Group A (Jilin)’ (Fig. 2)

^b^STs of Group B in ‘Group A (Jilin)’ (Fig. 2)

^c^Singletons in ‘Group A (Jilin)’ (Fig. 2)

In the nationwide analysis, ST369 was also a group founder with ST136, ST195, ST208, ST350 and ST540 as subgroup founders (Fig. 2B). The locations and distribution of Jilin STs in nationwide unrooted tree were like that in Jilin tree, but there was no nationwide subgroup founders ST136, ST350 and their downstream branches in Jilin tree. Subgroup founder ST208 had several variant STs, which located in three downstream branches. But in Jilin, as a predominant ST (14/89), ST208 did not occupy any variant and branches. This phenomenon suggested that ST208 in Jilin possibly was not a local isolate. It came from other domestic regions and induced an outbreak in our sample-taking hospital. Same to ST469, which was identified as a singleton in Jilin, but in nationwide, ST469 was variant of ST350, which was not detected in Jilin isolates (Fig. 2).

**Fig. 3:**
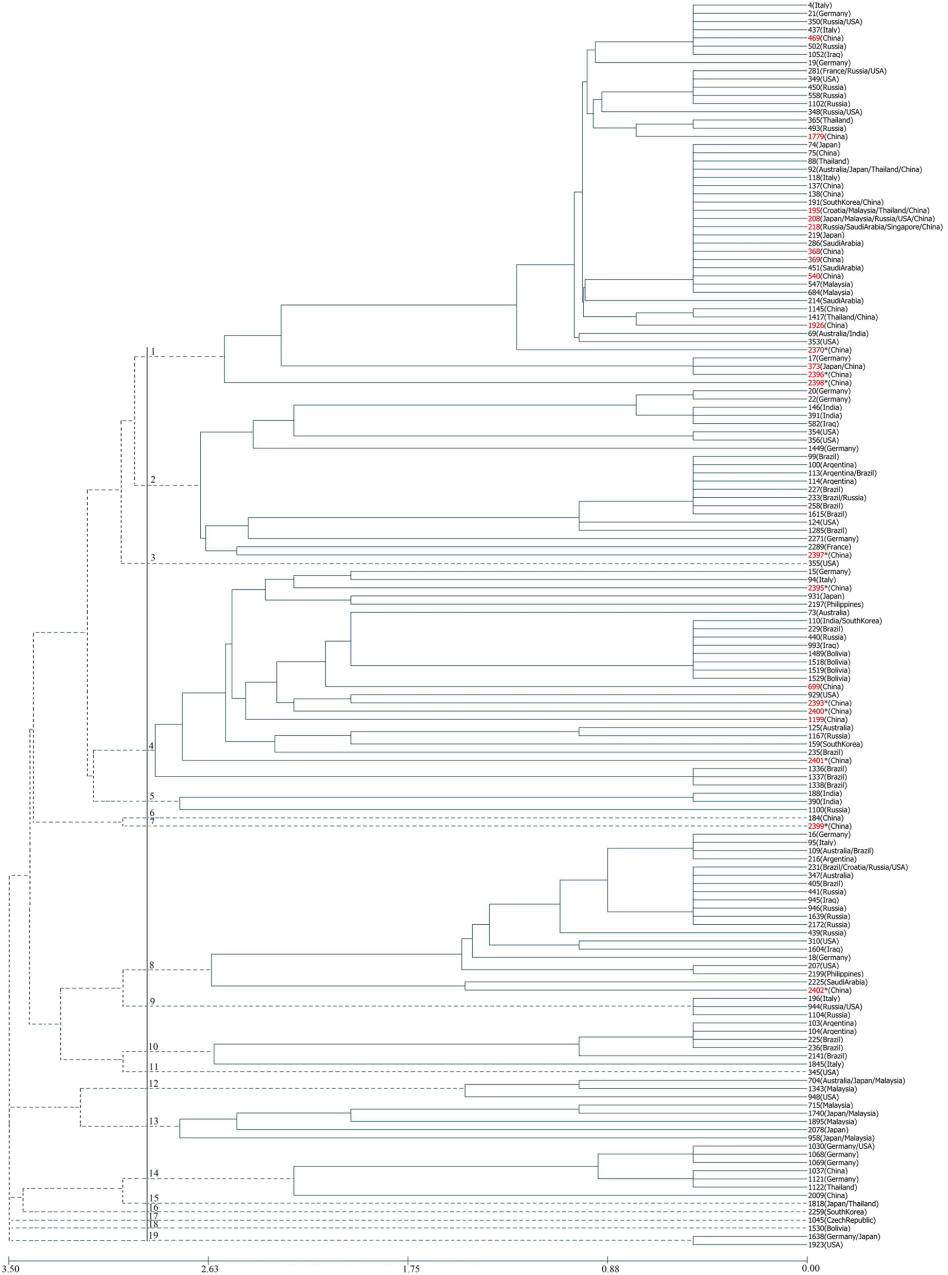
The evolutionary history inferred using the UPGMA method. The analysis involved 22 STs of this study from Jilin province which are shaded in red, another 10 predominant STs of China and selected 120 predominant STs from other countries. Jilin STs with * were newly discovered STs. Excepting ST2370, 2396 and 2398, that were distributed in the first group together with the predominant STs in China, the other newly discovered Jilin STs were distributed in other groups, which showed that these STs had closer relationship with foreign isolates

In worldwide analysis (Fig. 3), the majority of Jilin STs are classified in Group 1 along with predominant STs from Australia, France, Germany, Iraq, Italy, Japan, Malaysia, Russia, Saudi Arabia, Singapore, South Korea, Thailand, and the USA. And some Jilin STs are closely related to predominant STs from Australia, Bolivia, Brazil, India, South Korea, Russia, Iraq, and the USA in Group 4. ST2397 from Jilin had close relationship with French predominant ST2289 in Group 2. ST2402 is grouped in ‘Group 8’ along with predominant ST (ST2225) from Saudi Arabia. Among the novel STs in Jilin, only ST2399 without closely related strains in other countries, and it is divided into group 7 alone.

### Genetic relatedness and geographical distribution

**Fig. 4:**
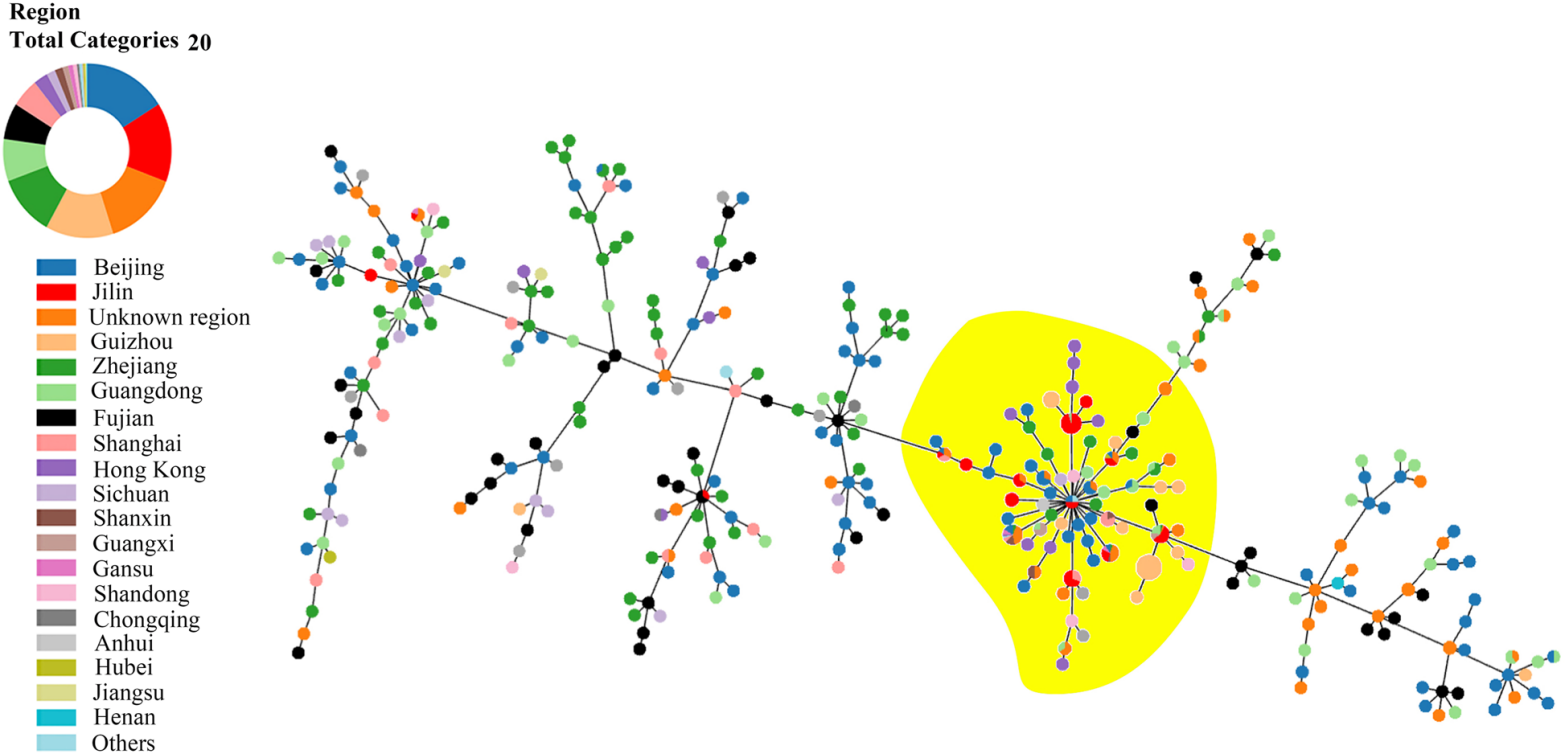
PHYLOViZ Online analysis showing the genetic relationship among China collection of sequence types (STs) of *A. baumannii*. Each dot in different color represents a distinct ST from different domestic regions. Jilin majorities were distributed in the yellow covered branches

By 19th of June 2021, total number of *A. baumannii* isolates was 3303 in MLST database, among them 653 were Chinese isolates.In China, 653 isolates distributed in 329 STs with frequency range 1–56, and major STs were ST1145 (*n* = 56), ST195 (*n* = 36), ST92 (*n* = 32), ST208 (*n* = 25), ST75 (*n* = 22), ST540 (*n* = 19), ST1417 (*n* = 17), ST138 (*n* = 10), ST191 (*n* = 10) and ST368 (*n* = 8). Jilin predominant STs shared same group founder ST369 with all STs of Guizhou (southeast China), and some sporadic isolates from other regions. But predominant STs in Guizhou were ST1646, ST1417, ST1145, ST2200, ST2208, ST2207, ST2204, ST1658, and ST1144 in Guizhou, which showed different origin and evolution trend from Jilin. The aggregation trend was not found in STs isolated from Zhejiang (east China), Shanghai (east China), Guangdong (south China), Fujian (south China), and Beijing (north China) (Fig. 4).

**Fig. 5:**
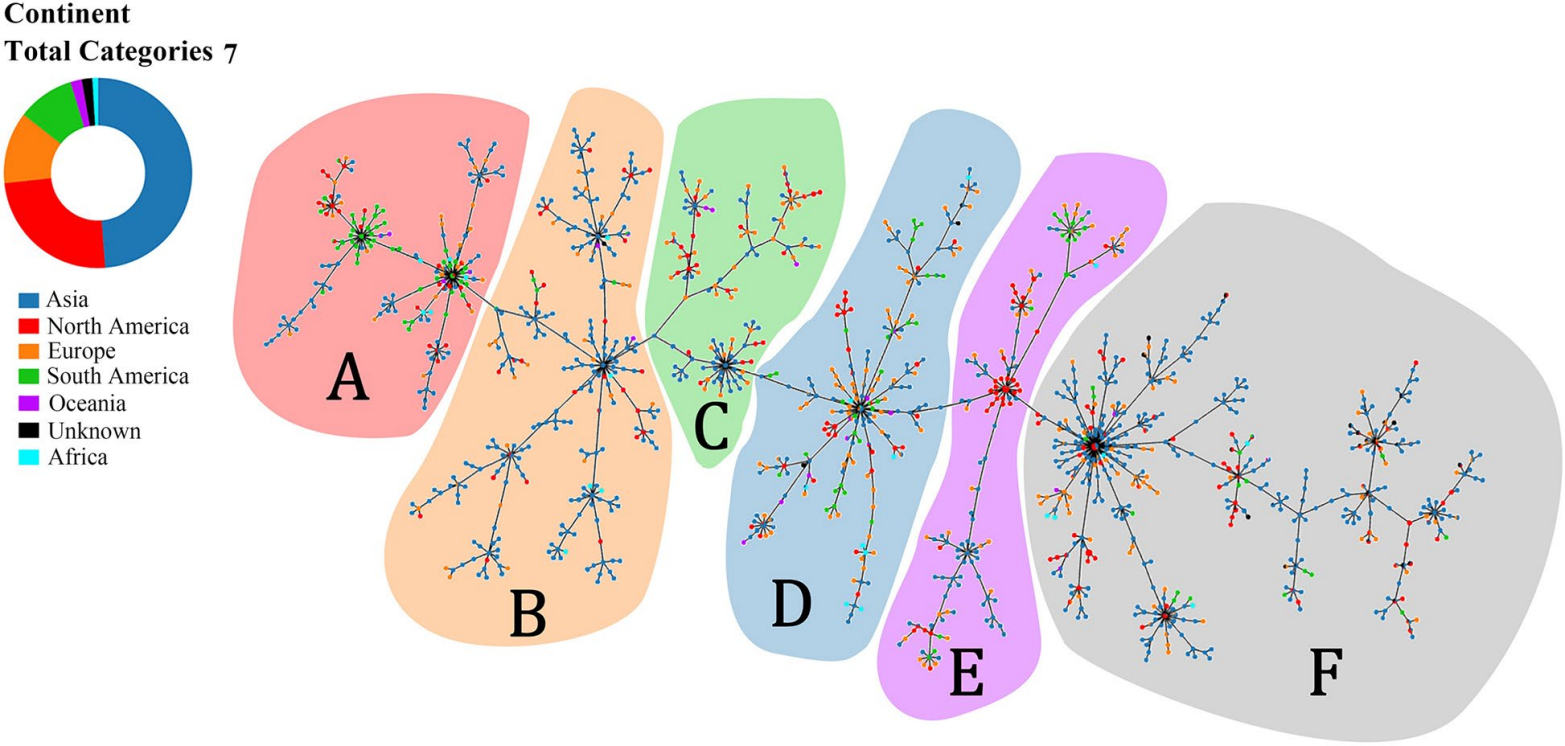
PHYLOViZ Online analysis shows the genetic relationship among continental collections of *A. baumannii* STs. Each dot represents a distinct ST. Group **A** (most of the STs from South America and Asia) is shaded in pink, Group **B** (most of the STs from Asia) is shaded in orange, Group **C** (most of the STs from Europe, Asia and North America) is shaded in green, Group **D** (most of the STs from Europe, Asia and South America) is shaded in blue, Group **E** (most of the STs from Asia, South America and North America) is shaded in purple, Group **F** (most of the STs from Asia, Europe and North America) is shaded in gray

**Fig. 6:**
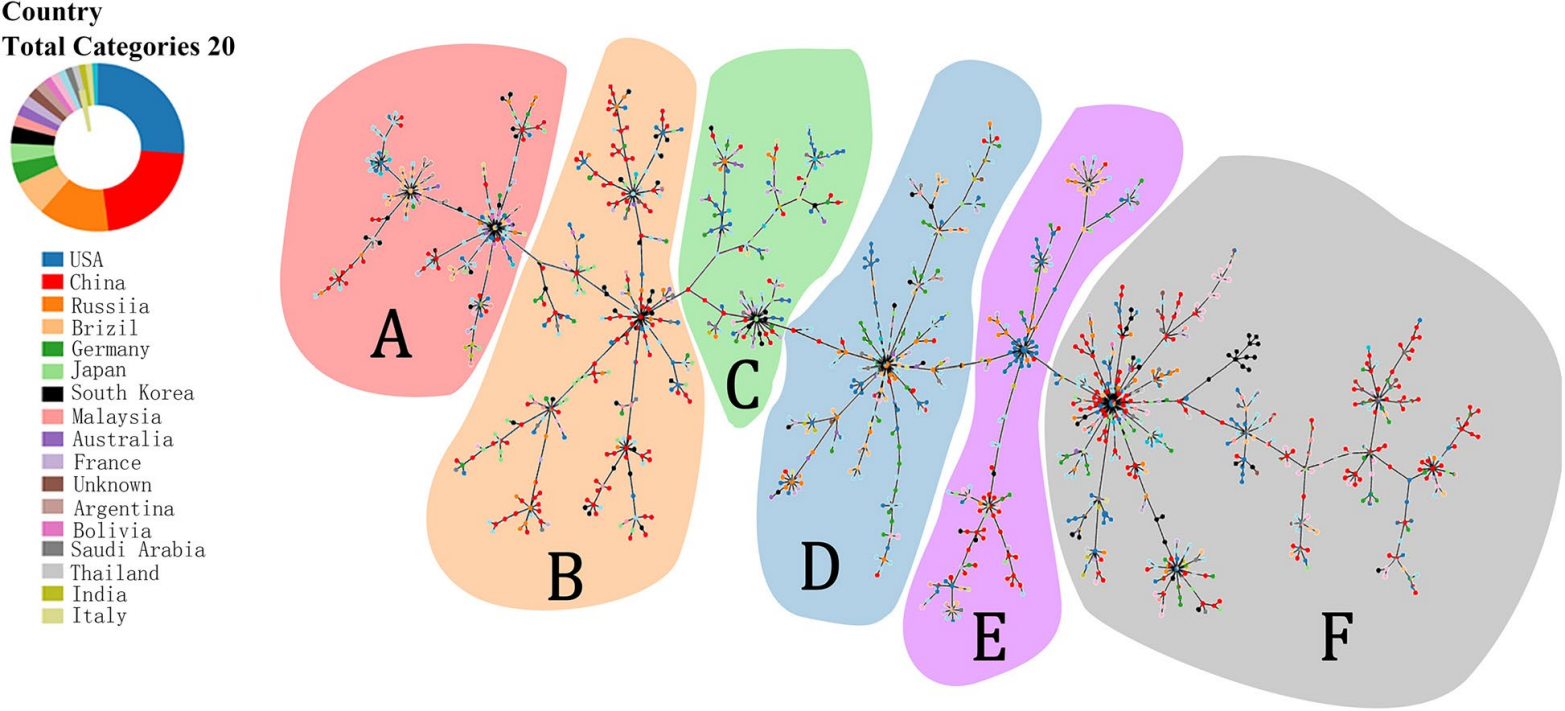
PHYLOViZ Online analysis shows the genetic relationship among different countries’ collection of *A. baumannii* STs. Each dot in different represents a distinct ST from different countries. The distributions of all STs are similar to that in Fig. 5, and all predominant STs of China were grouped in group **F** that mainly contained Asia isolates

If the analysis was performed worldwide, all STs were divided into six groups as shown in Fig. 5. All Chinese *A. baumannii* predominant STs belonged to Group F (Fig. 6).

## Discussion

In this study, all isolates came from single hospital, the overall diversity (STs/isolate) was about 0.247 (22/89) was not high, but the proportion of newly discoved ST and their origin were over our expectation. Ten novel STs were identified in 89 isolates, it could be said that the results of this study contributed newer STs to the MLST database. And many of newly discovered STs just with 1 isolate were regarded as singleton lacking relationship with previous discovered Chinese STs, but had closer relationship with foreign STs, which suggested that they are maybe the variant of foreign strains. The new immigrant with strong resistance, such as ST2933, should be paid more attention to continuously monitor and control its epidemiology because it will get more opportunities to spread and are more likely to become dominant strains in the future.

But isolates with domestic origin, such as ST208, which could be considered as a model to evaluate nosocomial infection of *A. baumannii* in this hospital. As a nation level major ST, ST208 had many variants and evolution branches (Fig. 2B), but in Jilin it had high occupation (14/89) without any variants and evolution branches (Fig. 2A), which suggested that it spread to this hospital and caused a nosocomial outbreak within short period. So, the samples being taken from single hospital was still helpful to epidemiological research, prevent and control *A. baumannii* infection. And the causative pathogen could be identified quickly, and infection prevention and control measures could be applied earlier if daily routine molecular typing is implemented [4].

The majority (81.1%) of Jilin STs belonged to CC92 group, which is the largest and most widely distributed *A. baumannii* clone in China [8]. According to the published articles about *A. baumannii* research in China [8, 11–17], epidemic STs in Jilin appeared earlier than non-epidemic STs in Jilin. But in Jilin isolates, some major Chinese STs in CC92 group were not founded, such as ST191 (Beijing and Hunan) [18, 19] and ST1145 (Guangdong) [20]. And the epidemic ST92, which was predicted to be the group founder of CC92 group, was never found in Jilin province too.

Multidrug resistance strains (MDR) of *A. baumannii* have increased because of the acquirement of mobile genetic elements such as transposons, plasmids, and integrons. Especially, carbapenems resistant *A. baumannii* (CR-Ab) is a worldwide problem because these strains are often resistant to all other commonly used antibiotics [21]. In the hospital, the antibiotic susceptibility of all Jilin isolates was routinely tested by K-B method. The tested antibiotics include amikacin, gentamicin, imipenem, meropenem, cefazolin, ceftazidime, cefotaxime, cefepime, aztreonam, ampicillin, piperacillin, amoxicillin/clavulanic acid, ampicillin/sulbactam, piperacillin/tazobactam, polymyxin, compound sulfonamide, chloramphenicol, ciprofloxacin, levofloxacin and tetracycline. Almost all isolates are resistant to cephalosporins, penicillins, gentamicin, and ciprofloxacin. As carbapenem is used as last line antibiotic treatment to eliminate infections with multidrug-resistant Gram-negative bacteria [21], we tested the carbapenems resistance in the lab again. At same time, resistance against tigecycline and colistin B were also tested again because they usually are members of final choices in China. The STs with earlier appearance and the stronger resistance, had more isolates, such as ST195 and ST540. Four isolates sensitive to all tested carbapenems distributed in ST373, ST2397, ST2399, and ST2402. STs, including ST373, ST2397, ST2399 and ST2402, which just occupied one isolates that sensitive to all tested carbapenems. Although the single isolates of ST373 appeared early in 2013, it was not found in following years because the sensitivity to carbapenems made it lost the chance of spreading. However, for single isolates of ST2397, ST2399 and ST2402, as singletons they appeared in 2017 and did not have links with local isolates. They were sporadic nation-cross spreading isolates because the result of evolutionary history by UPGMA method (Fig. 3) also showed they have close relationship with STs of other countries. The sensitivity to carbapenem also made them lost continuous dissemination. So, the reason for the small number of sensitive STs was gradually eliminated by carbapenems, while the reason for newly discovered STs was that new variant or new immigrant have not yet had time to reproduce.

Beside Jilin province, the patients also came from other domestic regions and neighboring countries. All strains were identified by Matrix Assisted Laser Desorption Ionization (MALDI)-Time of Light (TOF)-Mass Spectrometer (MS) and 16SrRNA encoding sequence analysis. However, there are still some strains that cannot be identified as *A. baumannii* or *A. colcaoaceticus*, which makes it impossible to include such strains in our study. Although the results of this study showed the unique molecular epidemiological characteristics of *A. baumannii* in Jilin Province, a single origin of isolates and a low number of isolates are also the shortcomings of this study. It will be more meaningful to obtain more isolates from more hospitals for similar study in the future.

## Conclusions

Overall, the antibiotics resistance of *A. baumannii* is very serious in Jilin Province, the proportion of bacteria resistant to at least one carbapenems antibiotic has exceeded 94% (84/89). Antibiotic resistance is a helpful factor for predominant STs spreading. The proportion of predominant STs in Jilin are different from some domestic regions, such as Guizhou, Beijing and Hunan, and suspicious nation cross new STs occupied high proportion in Jilin. Epidemiological research by MLST in single hospital is helpful to monitor and control nosocaomial infection of *A. baumannii* because newly discovered STs, genetic relatedness and geographical distribution reflected sufficient spreading clues of *A. baumannii*.

## Materials and methods

### Ethics statement

All bacteria are routinely collected for diagnosis in the Affiliated Hospital of Changchun University of Chinese Medicine. No extra samples and personal information about patients were used in the study. Therefore, a written personal informed consent and ethics committee approval were not required, and the study was in line with Chinese laws.

### Bacterial strains and growth conditions

Eighty-nine (*n* = 89) clinical isolates of *A. baumannii* were collected from the Affiliated Hospital of Changchun University of Chinese Medicine during 2013–2017. All the isolates were identified by automated VITEK2 and confirmed by 16SrRNA sequencing. For the preparation of DNA, bacteria were cultured overnight in Luria–Bertani (LB) broth at 37℃ in high containment facility, a biosafety level 2 facility at College of Basic Medical Science of Jilin University. Total DNA was extracted by *EasyPure®* Bacteria Genomic DNA Kit (TransGen Biotech) under the protocol and stored at -20℃ for further use.

### Antimicrobial susceptibility tests

All collected *A. baumannii* isolates were subjected to antimicrobial susceptibility test against ertapenem, imipenem, meropenem, tigecycline, and colistin B according broth dilution method following M100 Performance standards for antimicrobial susceptibility testing thirtieth edition of the Clinical and Laboratory Standards Institute (CLSI) [22]. *Escherichia coli* ATCC® 25,922 and *Pseudomonas aeruginosa* ATCC® 2785 were included as controls. Breakpoints of ertapenem (susceptible, ≤ 2 ug/L; intermediate, 4 ug/L; and resistant, ≥ 8 ug/L), imipenem (susceptible, ≤ 2 ug/L; intermediate, 4 ug/L; and resistant, ≥ 8 ug/L), meropenem (susceptible, ≤ 2 ug/L; intermediate, 4 ug/L; and resistant, ≥ 8 ug/L), tigecycline (susceptible, ≤ 4 ug/L; intermediate, 8 ug/L; and resistant, ≥ 16 ug/L), and colistin B (susceptible, ≤ 2 ug/L; and resistant, ≥ 4 ug/L) were the CLSI guidelines clinical breakpoints for *Acinetobacter* spp. The association between STs and the antimicrobial susceptibility among the study population was carried out using Fisher’s precision probability test. And all antibiotics were the products of Sigma.

### Multi locus sequence typing (MLST)

MLST was carried out according to the ‘Oxford’ scheme [23]. Primers in MLST site (https://pubmlst.org/primers-used-mlst-acinetobacter-baumannii-complex-oxford-scheme) were used to amplify *gltA*, *gyrB*, *gdhB*, *recA*, *cpn60*, *gpi*, and *rpoD*, which are conserved housekeeping genes in MLST analysis (Table 4). (https://pubmlst.org/primers-used-mlst-acinetobacter-baumannii-complex-oxford-scheme). PCR amplification, sequence analysis and determination of ST for each isolate were carried out according to online *A. baumannii* typing protocols (https://pubmlst.org/bigsdb?db=pubmlst_abaumannii_seqdef). The PCR amplicons were double pass sequenced using commercial SANGER sequencing services (Comate Bioscience Co., Ltd.). Novel sequence types (STs) were submitted to the *A. baumannii* MLST Database.

**Table 4:** Primers used for MLST of *A. baumannii* complex (Oxford scheme)

Locus	Primer	Sequences	Amplicon size (bp)	Usage
***gltA***	Citrato F1	AAT TTA CAG TGG CAC ATT AGG TCC C	722	amp/seq
	Citrato R12	GCA GAG ATA CCA GCA GAG ATA CAC G		amp/seq
***gyrB***	gyrB_F	TGA AGG CGG CTT ATC TGA GT	594	amp/seq
	gyrB_R	GCT GGG TCT TTT TCC TGA CA		amp/seq
***gdhB***	GDHB 1F	GCT ACT TTT ATG CAA CAG AGC C	774	amp
	GDH SEC F	ACC ACA TGC TTT GTT ATG		seq
	GDHB 775R	GTT GAG TTG GCG TAT GTT GTG C		amp
	GDH SEC R	GTT GGC GTA TGT TGT GC		seq
***recA***	RA1	CCT GAA TCT TCY GGT AAA AC	425	amp/seq
	RA2	GTT TCT GGG CTG CCA AAC ATT AC		amp/seq
***cpn60***	cpn60_F	GGT GCT CAA CTT GTT CGT GA	640	amp/seq
	cpn60_R	CAC CGA AAC CAG GAG CTT TA		amp/seq
***gpi***	gpi_F	GAA ATT TCC GGA GCT CAC AA	456	amp/seq
	gpi_R	TCA GGA GCA ATA CCC CAC TC		amp/seq
***rpoD***	rpoD-F	ACC CGT GAA GGT GAA ATC AG	672	amp/seq
	rpoD-R	TTC AGC TGG AGC TTT AGC AAT		amp/seq

### Phylogenetic analysis

The relatedness analysis was performed by online goeBURST and eBURST under the criteria of single locus variant (SLV) as both goeBURST and eBURST allow unrooted tree-based relationship representation of the analyzed isolates ( http://eburst.mlst.net/) [24, 25]. The criteria ensures that even single locus variation of housekeeping genes will be displayed in unrooted tree. Factors such as the number of alleles, variable sites in each allele, frequency of single nucleotide polymorphism (SNPs) were also considered in the algorithm of two online methods. As the distance between original genotype and variant usually suggests the degree of variation, the distance from triple locus variants (TLVs) to original genotype is longer than that from SLVs and double locus variants (DLVs) to original genotype.

Nucleotide sequence diversity (π) was calculated using DNAsp V5.1 software [26]. All Chinese STs in including Jilin isolates were compared to isolates from other countries by PHYLOViZ Online of MLST site (http://pubmlst.org/bpseudomallei), which is a flexible tool for relationship and regional characteristic analysis [27].

To further determine the molecular evolutionary relationship between Chinese STs and prevalent STs in other countries, 22 Jilin STs, another 10 Chinese epidemic STs (isolates ≥ 8), and 120 epidemic STs (isolates ≥ 2) from other countries were selected and analyzed by Unweighted Pair Group Method (UPGM) of PHYLOViZ program [28, 29]. In other article, this analysis also was performed by Arithmetic average method in molecular evolutionary genetic analysis (MEGA) software [30, 31]. The topology and grouping of all STs were displayed on constructed boot-strapped phylogenetic tree.

## Data Availability

All data generated or analyzed during this study are included in this published article and all isolates ID are listed in Table 2. And the datasets generated during and/or analyses during the current study are available in the PubMLST (*Acinetobacter baumannii* database: https://pubmlst.org/bigsdb?db=pubmlst_abaumannii_isolates).
